# Evaluation of the protective effects of green tea for ischemia–reperfusion injury in rat ovary tissue: an experimental study

**DOI:** 10.1590/acb415526

**Published:** 2026-09-21

**Authors:** Hatice Yilmaz Dogru, Akgul Arici, Emine Tekingunduz, Cuma Tasin

**Affiliations:** 1 Mersin City Training and Research Hospital – Department of Obstetrics and Gynecology – Mersin, Turkey.; 2 Tokat Gaziosmanpasa University – School of Medicine – Department of Pathology – Tokat, Turkey.; 3 Mersin City Training and Research Hospital – Department of Pathology – Mersin, Turkey.

**Keywords:** Tea, Ovary, Apoptosis, Wounds and Injuries

## Abstract

**Purpose:**

To investigate the protective effects of green tea extract on ovarian ischemia–reperfusion injury in a rat model using histopathological and immunohistochemical analyses.

**Methods:**

Twenty-one female Wistar–Albino rats were randomly divided into three groups (n = 7 each): sham, saline-treated ischemia–reperfusion, and green tea extract-treated ischemia–reperfusion. Ovarian torsion was induced by 720° clockwise rotation of the adnexa for three hours, followed by three hours of reperfusion. Green tea extract (200 mg/kg) or saline was administered intraperitoneally immediately before reperfusion. Ovarian tissues were evaluated histopathologically for congestion, hemorrhage, edema, polymorphonuclear leukocyte infiltration, and follicular degeneration. Bax and caspase-3 expression levels were assessed immunohistochemically.

**Results:**

The saline group demonstrated significantly increased congestion, edema, follicular degeneration, and total injury scores compared with the sham group. Green tea extract administration significantly reduced congestion, edema, and follicular degeneration compared with the saline group, indicating partial histopathological protection against ovarian ischemia–reperfusion injury. However, Bax and caspase immunoreactivity remained elevated in the treatment group.

**Conclusion:**

Green tea extract attenuated histopathological ovarian damage following ischemia–reperfusion injury, suggesting a protective effect against ovarian tissue injury. Although treatment was associated with increased expression of apoptotic markers, the overall findings support the potential of green tea-derived antioxidants as a therapeutic strategy for ovarian ischemia–reperfusion injury.

## Introduction

Ovarian torsion is one of the most important gynecological emergencies requiring prompt diagnosis and surgical intervention to preserve ovarian viability and reproductive function. It occurs as a result of partial or complete rotation of the ovary and vascular pedicle around its supporting ligaments, leading to impaired venous and lymphatic drainage followed by arterial obstruction and tissue ischemia^1^. Delayed diagnosis or prolonged ischemia may result in ovarian necrosis, infertility, endocrine dysfunction, and loss of ovarian reserve^2^. Although detorsion and restoration of blood flow are essential for ovarian salvage, reperfusion itself may paradoxically aggravate tissue injury through complex biochemical and inflammatory mechanisms known as ischemia–reperfusion (I/R) injury^3^.

The pathophysiology of ovarian I/R injury involves excessive production of reactive oxygen species (ROS) during the reperfusion phase^4^. Under ischemic conditions, depletion of intracellular ATP, mitochondrial dysfunction, calcium overload, and endothelial injury occur within ovarian tissue. Subsequent reoxygenation following reperfusion results in abrupt generation of superoxide radicals, hydrogen peroxide, hydroxyl radicals, and reactive nitrogen species, leading to lipid peroxidation, protein oxidation, DNA damage, and cellular apoptosis^4,5^. Furthermore, activation of neutrophils and inflammatory cytokines contributes to microvascular dysfunction, increased vascular permeability, edema formation, and follicular degeneration^3^. These pathological processes may ultimately impair ovarian reserve and fertility potential.

Oxidative stress has been identified as a major contributor to ovarian follicular injury and reproductive dysfunction^5^. Ovarian follicles, particularly granulosa cells and oocytes, are highly vulnerable to oxidative damage because of their intense metabolic activity and mitochondrial dependence^6^. Previous experimental studies have demonstrated that ovarian I/R injury is associated with histopathological findings such as vascular congestion, hemorrhage, edema, inflammatory infiltration, follicular atresia, and apoptotic cell death^7^. Therefore, prevention of oxidative stress-induced ovarian injury has become an important focus of experimental research.

In recent years, antioxidant agents have attracted considerable attention as potential therapeutic strategies against I/R injury. Several experimental studies have investigated the protective effects of antioxidant compounds including melatonin, curcumin, resveratrol, erythropoietin, N-acetylcysteine, and vitamin E in ovarian torsion models^7-9^. These agents have been shown to reduce oxidative stress, suppress inflammatory responses, preserve follicular morphology, and improve ovarian tissue integrity following reperfusion. However, no universally accepted pharmacological treatment currently exists for prevention of ovarian I/R injury.

Green tea, derived from the leaves of *Camellia sinensis*, is one of the most widely consumed beverages worldwide and is rich in polyphenolic catechins with potent antioxidant and anti-inflammatory properties^10^. The major biologically active catechins in green tea include epigallocatechin gallate, epigallocatechin, epicatechin gallate, and epicatechin^11^. These compounds have been shown to scavenge free radicals, inhibit lipid peroxidation, modulate inflammatory pathways, and enhance endogenous antioxidant enzyme activity^11,12^. Experimental studies in different organ systems have demonstrated that green tea polyphenols may protect against I/R injury in the liver, kidney, heart, and brain tissues^13,14^. In addition, green tea catechins have been reported to influence apoptotic signaling pathways through regulation of Bax, Bcl-2, and caspase proteins^15^.

Apoptosis plays a central role in ovarian I/R injury. Bax is a proapoptotic member of the Bcl-2 protein family that promotes mitochondrial membrane permeabilization and activation of downstream apoptotic cascades^16^. Caspases, particularly caspase-3, function as critical executioner enzymes responsible for programmed cell death^17^. Increased expression of Bax and caspase proteins has been associated with oxidative stress-mediated ovarian follicular injury and granulosa cell apoptosis during I/R processes^16,17^. Therefore, evaluation of these apoptotic markers may provide important information regarding the cellular effects of antioxidant therapies.

Although the antioxidant properties of green tea have been extensively investigated in various experimental conditions, limited data are available regarding its effects on ovarian I/R injury. Therefore, the present experimental study aimed to investigate the potential protective effects of green tea extract on ovarian tissue injury in a rat model of I/R using histopathological and immunohistochemical analyses. In addition, apoptotic activity was evaluated through assessment of Bax and caspase expression levels in ovarian tissue.

## Methods

### Chemicals

Ketamine and xylazine were obtained from Alfasan International B.V. (Woerden, Netherlands). Hematoxylin Harris and eosin Y (1% alcoholic) were purchased from Atom Scientific LTD (Manchester, United Kingdom). Bouin’s solution was supplied by Tek-Path Medikal (Izmir, Turkey). Green tea extract used in the experimental protocol was prepared for intraperitoneal administration under sterile laboratory conditions.

### Animals

The experimental study was conducted following approval from the Local Ethics Committee for Animal Experiments of Tokat Gaziosmanpaşa University (Approval No.: 2013-HADYEK-12). A total of 21 adult female Wistar–Albino rats weighing 250–300 g were included in the study. The animals were obtained from the Experimental Medicine Research Unit of Tokat Gaziosmanpaşa University. All experiments were conducted in Tokat Gaziosmanpasa University Experimental Medicine Research Unit laboratories, Tokat, Turkey.

All rats were housed under standardized laboratory conditions with a 12-hour light/dark cycle, controlled room temperature (20–24°C), and relative humidity of 40–50%. Animals were maintained in polycarbonate cages with free access to standard laboratory chow and tap water *ad libitum*. Cage cleaning and environmental monitoring were performed regularly throughout the study period in accordance with institutional guidelines for laboratory animal care and welfare.

### Experimental design

The rats were randomly assigned into three experimental groups using a computer-generated randomization method, with seven rats included in each group:

- Sham group: under general anesthesia, a midline abdominal incision was performed, and bilateral ovaries were exposed without induction of adnexal torsion. Intraperitoneal saline was administered;
- Saline group: following induction of general anesthesia, adnexal torsion involving the ovary and fallopian tube was created by rotating the adnexa clockwise by 720°. The adnexal torsion was fixed to the anterior abdominal wall using a 4/0 silk suture to maintain the torsion. Ovarian ischemia was maintained for three hours, followed by detorsion and a subsequent three-hour reperfusion period. Immediately before reperfusion, 1-mL intraperitoneal saline was administered;
- Green tea extract group: after induction of general anesthesia, a 720° clockwise adnexal torsion including the ovary and fallopian tube was established in the same manner as in saline group. The adnexal torsion was fixed to the anterior abdominal wall using 4/0 silk suture. Following three hours of ischemia, detorsion was performed and reperfusion was allowed for three hours. Immediately before reperfusion, green tea extract at a dose of 200 mg/kg was administered intraperitoneally.

All animals were fasted for 12 hours prior to the surgical procedure while maintaining free access to water. General anesthesia was induced using intraperitoneal ketamine hydrochloride at a dose of 50 mg/kg combined with xylazine hydrochloride at a dose of 10 mg/kg. Adequate depth of anesthesia was confirmed by the absence of withdrawal reflexes before initiation of the surgical procedure.

Under general anesthesia and sterile surgical conditions, the abdominal area was shaved and disinfected with antiseptic solution. A lower midline abdominal incision was then performed to expose the pelvic organs and adnexal structures. All surgical interventions and animal monitoring were carried out under aseptic conditions by the same research team to ensure procedural standardization among experimental groups.

### Tissue collection and histopathological examination

At the end of the experimental period, euthanasia was performed under anesthesia induced by intraperitoneal ketamine (90 mg/kg) and xylazine (10 mg/kg). Bilateral ovarian tissues were carefully dissected and collected for histopathological and immunohistochemical analyses.

Ovarian tissue samples were fixed in Bouin’s solution for 24 hours, dehydrated through graded alcohol series, cleared in xylene, and embedded in paraffin blocks. Paraffin-embedded tissues were sectioned into 5-μm-thick slices and stained with hematoxylin and eosin (H&E) for histopathological evaluation under light microscopy.

Histopathological evaluation was performed to assess ovarian tissue injury associated with I/R damage and the potential protective effects of treatment. Tissue injury was graded using a semiquantitative scoring system previously described in experimental ovarian I/R studies by Hizal et al.7 and modified according to the severity of histopathological findings. Each parameter was scored on a scale from 0 to 3, where 0 indicated no pathological damage, 1 mild damage, 2 moderate damage, and 3 severe damage^7,18^.

The evaluated histopathological parameters included vascular congestion, hemorrhage, edema, polymorphonuclear leukocyte (PMNL) infiltration, and follicular degeneration. Follicular degeneration was assessed according to structural alterations in oocytes and granulosa cells together with reduction or loss of primordial, primary, secondary, and tertiary follicles. Vascular congestion was defined as increased blood accumulation within ovarian vessels, whereas hemorrhage was characterized by extravasation of erythrocytes into the ovarian stroma. PMNL infiltration was evaluated as an indicator of inflammatory response, and interstitial edema was assessed according to tissue swelling and extracellular fluid accumulation.

Each parameter was scored individually, and total injury scores and mean injury scores were subsequently calculated for each specimen. Histopathological examinations were performed by blinded observers under light microscopy using standard morphological criteria.

### Immunohistochemical analysis

Immunohistochemical staining was performed to evaluate apoptotic activity in ovarian tissue through the expression of Bax and caspase proteins. Paraffin-embedded ovarian tissue sections of 5-μm thickness were deparaffinized in xylene (Merck KGaA, Darmstadt, Germany) and rehydrated through graded ethanol solutions (Merck KGaA, Darmstadt, Germany). Antigen retrieval was carried out using citrate buffer solution (pH 6.0; Thermo Fisher Scientific, Waltham, MA, United States of America) under heat treatment in a microwave oven. Endogenous peroxidase activity was blocked with 3% hydrogen peroxide solution (Sigma-Aldrich, St. Louis, MO, United States of America) prior to antibody incubation.

The tissue sections were incubated with primary antibodies against Bax (Rabbit polyclonal anti-Bax antibody; Abcam, Cambridge, United Kingdom) and caspase-3 (Rabbit polyclonal anti-caspase-3 antibody; Abcam, Cambridge, United Kingdom) according to the manufacturer’s recommended protocols. Following primary antibody incubation, sections were treated with horseradish peroxidase-conjugated secondary antibodies using a commercially available immunohistochemical detection kit (Thermo Fisher Scientific, Waltham, MA, United States of America). Immunoreactivity was visualized using 3,3’-diaminobenzidine (DAB) chromogen solution (Dako, Glostrup, Denmark), and the sections were counterstained with hematoxylin (Atom Scientific LTD, Manchester, United Kingdom).

Immunohistochemical evaluation was performed under light microscopy (Olympus BX51, Olympus Corporation, Tokyo, Japan). Bax and caspase expression levels were assessed according to the intensity and distribution of cytoplasmic staining in ovarian stromal and follicular cells using a semiquantitative immunohistochemical scoring system previously described by Hsu et al.^19^ and modified for ovarian tissue analysis^16^. Staining intensity was graded as follows:

- 0: no staining;
- 1: weak staining;
- 2: moderate staining;
- 3: strong staining.

The percentage of positively stained cells was also evaluated in randomly selected high-power microscopic fields, and mean immunoreactivity scores were calculated for each specimen.

Bax expression was evaluated as an indicator of proapoptotic activity associated with mitochondrial apoptotic pathways, whereas caspase expression was used to assess activation of apoptosis-related cellular mechanisms. Increased immunoreactivity for Bax and caspase was interpreted as enhanced apoptotic response within ovarian tissue. All immunohistochemical evaluations were performed by blinded observers to minimize assessment bias.

## Results

A total of 21 rats were included in the study, with seven rats allocated to each experimental group: sham, saline, and green tea extract.

### Histopathological findings

**Figure 1:**
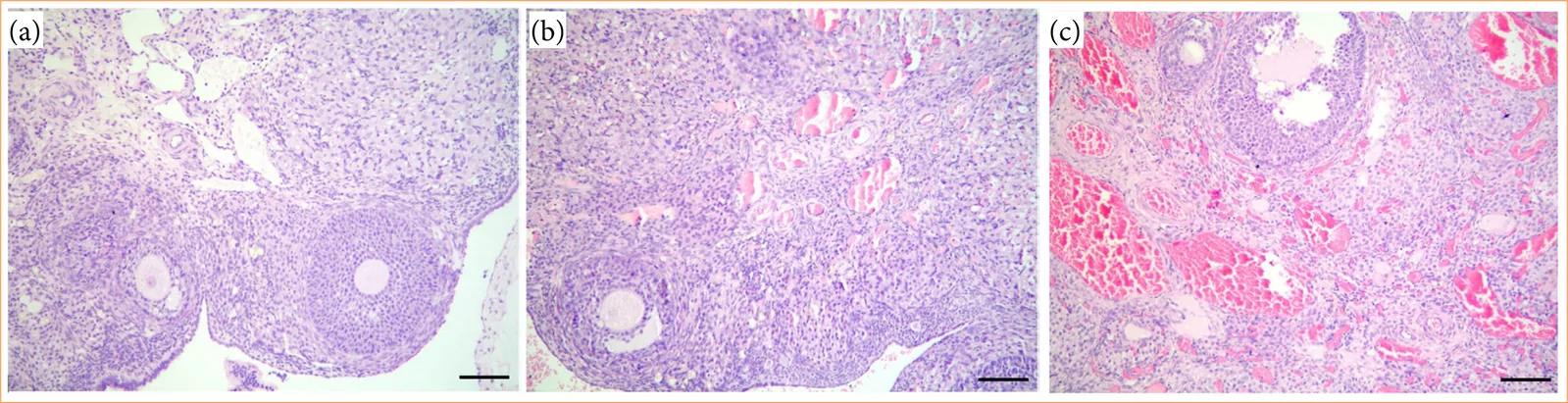
Ovary tissue image in sham group. Histological sections of rat ovary tissues staining by hematoxylin and eosin. (a) Sham group; (b) saline group. Arrows show severe congestion, hemorrhage, and follicular degeneration in focal areas of the ovarian tissue. (c) Green tea extract group. Arrows show moderate congestion and hemorrhagic findings in the ovarian tissue. Seven animals were included in each group, and six testis sections from each of five animals per group were assessed (n = 21). Scale bars: 50 μm. Magnification: 100x.

Source: Elaborated by the authors.

Histopathological injury scores are summarized in Table 1, and histopathological sections in all groups are shown in Fig. 1.

**Table 1:** Histopathological injury scores^<tfn href="tfn02">#</tfn>^.

	Sham group	Saline group	Green tea extract group	*p* -value
Congestion	0.14 ± 0.37	2.28 ± 0.95	1 ± 0.81	0.002*
Hemorrhage	0.14 ± 0.37	1 ± 1.15	1.28 ± 1.11	0.076
Edema	0	0.85 ± 0.37	0.42 ± 0.53	0.007*
PMNL infiltration	0	0.14 ± 0.37	0	0.368
Follicle degeneration	0	0.57 ± 0.53	0.14 ± 0.37	0.039*
Injury score	0.28 ± 0.48	4.85 ± 2.34	2.85 ± 1.77	0.002*
Mean injury score	0.04 ± 0.06	0.69 ± 0.33	0.4 ± 0.25	0.002*

* *p* < 0.05; Kruskal-Wallis’ test; Mann-Whitney’s U test; PMNL: polymorphonuclear leukocyte;

# intragroup comparisons related to congestion: sham

(S)- saline (SL): *p* = 0.001; S- green tea extract group (GT): *p* = 0.053; SL-GT: *p* = 0.038; intragroup comparisons related to hemorrhage: S-SL: *p* = 0.165; S-GT: *p* = 0.053; SL-GT: *p* = 0.620; intragroup comparisons related to edema: S-SL: *p* = 0.004; S-GT: *p* = 0.209; SL-GT: *p* = 0.209; intragroup comparisons related to PMNL infiltration: S-SL: *p* = 0.710; S-GT: *p* > 0.05; SL-GT: *p* = 0.710; intragroup comparisons related to follicle degeneration: S-SL: *p* = 0.073; S-GT: *p* = 0.710; SL-GT: *p* = 0.209; intragroup comparisons related to injury score: S-SL: *p* = 0.001; S-GT: *p* = 0.007; SL-GT: *p* = 0.165; intragroup comparisons related to mean injury score: S-SL: *p* = 0.001; S-GT: *p* = 0.007; SL-GT: *p* = 0.165. Source: Elaborated by the authors.

The saline group demonstrated significantly higher tissue injury compared with the sham group in several parameters, including congestion, edema, total injury score, and mean injury score.

Congestion scores differed significantly among the groups (*p* = 0.002). The saline group exhibited the highest congestion score, whereas the green tea extract group showed a lower score, suggesting partial attenuation of vascular congestion following green tea extract administration. Pairwise comparisons demonstrated significant differences between the sham and saline groups (*p* = 0.001) and between the saline and green tea groups (*p* = 0.038).

Edema scores were also significantly different among groups (*p* = 0.007). No edema was observed in the sham group, while the saline group demonstrated marked edema formation. The green tea extract group exhibited reduced edema scores (0.42 ± 0.53) compared with the saline group. Pairwise analysis revealed a significant difference between the sham and saline groups (*p* = 0.004).

Follicular degeneration showed a statistically significant overall difference between groups (*p* = 0.039). Degeneration scores were the highest in the saline group, whereas lower values were observed in the green tea extract group, and no degeneration was detected in the sham group.

Although hemorrhage scores were higher in the saline and green tea extract groups compared with the sham group, the difference did not reach statistical significance (*p* = 0.076). Similarly, PMNL infiltration scores were low in all groups, and no statistically significant difference was observed (*p* = 0.368).

The overall injury score demonstrated significant intergroup differences (*p* = 0.002). The saline group exhibited the highest injury score, whereas the green tea extract group showed a lower injury score. The sham group had the lowest injury score. Pairwise comparisons revealed significant differences between sham and saline groups (*p* = 0.001) and between sham and green tea extract groups (*p* = 0.007), while the difference between saline and green tea extract groups was not statistically significant (*p* = 0.165).

Similarly, mean injury scores differed significantly among groups (*p* = 0.002). The saline group had the highest mean injury score, while lower values were observed in the green tea extract group and sham group. Significant differences were identified between the sham and saline groups (*p* = 0.001) and between the sham and green tea extract groups (*p* = 0.007).

### Immunohistochemical findings

Immunohistochemical analysis scores for caspase and Bax expression are presented in Table 2. Significant intergroup differences were observed for both caspase and Bax immunoreactivity (*p* = 0.001 for both parameters).

**Table 2:** Immunohistochemical analysis scores<tfn href="tfn04">#</tfn>.

	Sham group	Saline group	Green tea extract group	*p* -value*
Caspase	156.1 ± 30.37	215.5 ± 16.18	198.33 ± 7.22	0.001
Bax	103.19 ± 3.17	123.6 ± 3.99	109.16 ± 3.45	0.001

* *p* < 0.05; Kruskal-Wallis’ test;

Mann-Whitney’s U test;

# intragroup comparisons related to caspase: sham (S)- saline (SL): *p* = 0.004;

S- green tea extract group (GT): *p* = 0.008; SL-GT: *p* = 0.016; intragroup comparisons related to Bax: S-SL: *p* = 0.004; S-GT: *p* = 0.019; SL-GT: *p* = 0.003. Source: Elaborated by the authors.

Caspase expression levels were significantly higher in the saline-treated I/R group compared with the sham group (*p* = 0.004). Although the green tea extract group demonstrated lower caspase expression than the saline group, caspase immunoreactivity remained significantly elevated compared with the sham group (*p* = 0.008). Pairwise comparisons revealed significant differences between saline and green tea extract groups (*p* = 0.016).

Similarly, Bax expression differed significantly among the experimental groups (*p* = 0.001). The highest Bax immunoreactivity was observed in the saline group compared to sham group (*p* = 0.004). Higher Bax expression levels were detected in the green tea extract group than in sham group (*p* = 0.019). Additionally, pairwise analysis demonstrated statistically significant differences between the saline and green tea extract groups (*p* = 0.003).

## Discussion

The main findings of the current study demonstrated that ovarian I/R injury resulted in significant histopathological damage characterized by increased vascular congestion, edema, follicular degeneration, and elevated total injury scores in the saline-treated group. Administration of green tea extract attenuated vascular congestion, edema, and follicular degeneration compared with the untreated I/R group. However, despite these histopathological improvements, Bax and caspase immunoreactivity remained significantly elevated in the treatment group.

Oxidative stress is considered one of the principal mechanisms underlying I/R injury^4,5^. During ischemia, depletion of intracellular ATP, mitochondrial dysfunction, endothelial damage, and ionic imbalance occur within ovarian tissue. Reperfusion subsequently induces excessive production of ROS, including superoxide radicals, hydrogen peroxide, and hydroxyl radicals, leading to lipid peroxidation, DNA damage, protein oxidation, and cellular injury^4,12^. Ovarian follicles are particularly susceptible to oxidative damage because granulosa cells and oocytes possess high metabolic activity and mitochondrial dependence^5,6^. Therefore, attenuation of oxidative stress may contribute substantially to preserve ovarian morphology and reproductive potential.

The significant increase in congestion and edema observed in the saline-treated I/R group confirms successful induction of ovarian tissue injury in the present model. Similar histopathological findings have been reported in previous experimental studies evaluating ovarian torsion and reperfusion injury. Hizal et al.^7^ demonstrated severe vascular congestion, edema, hemorrhage, and follicular degeneration following ovarian I/R injury in rats. Likewise, Yilmaz et al.^18^ reported marked inflammatory and degenerative ovarian changes associated with ischemic injury. The findings of the current study are therefore consistent with previously established experimental ovarian torsion models.

Green tea extract administration resulted in significant reduction of congestion and edema scores compared with the saline group, suggesting partial protection against microvascular and inflammatory injury. Green tea contains biologically active catechins, particularly epigallocatechin gallate, which possess potent antioxidant and anti-inflammatory properties^10,11^. These compounds can scavenge ROS, inhibit lipid peroxidation, suppress neutrophil activation, and enhance endogenous antioxidant enzyme activity such as superoxide dismutase and catalase^11-13^. Experimental studies in different organ systems have demonstrated protective effects of green tea polyphenols against I/R injury in the liver, kidney, heart, and brain.^13,14^

Follicular degeneration was also significantly reduced in the green tea extract group compared with the saline-treated I/R group. Preservation of follicular integrity is particularly important because ovarian reserve and fertility potential depend largely on the maintenance of viable follicles and granulosa cells^5,6^. Oxidative stress-induced follicular degeneration and oocyte damage have been associated with impaired reproductive function following ovarian torsion^20^. Previous experimental studies evaluating antioxidant therapies such as melatonin, curcumin, and N-acetylcysteine similarly demonstrated improved follicular preservation after ovarian I/R injury^7-9^. The current findings support the growing evidence suggesting that antioxidant-based therapies may have beneficial effects in protecting ovarian tissue during reperfusion injury.

Despite histopathological improvement, Bax and caspase expression levels remained significantly elevated in the green tea extract group. Bax is a proapoptotic member of the Bcl-2 family that promotes mitochondrial membrane permeabilization and activation of downstream apoptotic cascades^16^. Caspases are critical executioner enzymes involved in programmed cell death^17^. Increased Bax and caspase expression generally indicate activation of apoptotic pathways in response to cellular stress and oxidative injury^16,17^.

The elevation of apoptotic markers in the green tea extract group may initially appear contradictory to the reduction in histopathological injury. However, apoptosis and necrosis represent distinct mechanisms of cell death. Necrosis causes uncontrolled cellular destruction and inflammatory amplification, whereas apoptosis is a regulated process allowing selective elimination of damaged cells with minimal inflammatory response^21^. Therefore, it is possible that green tea extract reduced widespread necrotic injury while facilitating controlled apoptotic removal of irreversibly damaged ovarian cells. Such regulated apoptotic activity may contribute to preserve the overall tissue architecture and limit secondary inflammatory injury.

Another possible explanation involves the dual antioxidant and prooxidant effects of green tea catechins. Although catechins generally function as antioxidants under physiological conditions, higher concentrations of epigallocatechin gallate may exert prooxidant or apoptosis-inducing effects depending on dose, tissue environment, and oxidative state^15,22^. Bao et al.^22^ reported that green tea polyphenols may activate apoptotic signaling pathways through modulation of Bax and caspase activity. Similarly, Mia et al.^15^ demonstrated that tea polyphenols can exhibit both antioxidant and prooxidant biological behavior depending on experimental conditions. Therefore, the increased Bax and caspase immunoreactivity observed in the current study may reflect activation of regulated apoptotic signaling secondary to catechin-mediated cellular responses.

The discrepancy between histopathological improvement and increased apoptotic marker expression may also be related to the timing of tissue sampling, as apoptotic signaling is often activated during early reperfusion before structural recovery becomes evident^4^. Consequently, elevated Bax and caspase expression may represent transient cellular adaptation rather than irreversible tissue injury.

The protective effects of green tea extract observed in the present study also parallel findings reported for melatonin, one of the most extensively investigated antioxidants in experimental ovarian I/R models. Consistent with the present findings, melatonin has consistently been shown to preserve ovarian tissue architecture and attenuate oxidative injury through enhancement of endogenous antioxidant defenses. Recently, Monteiro et al.^23^ demonstrated that melatonin significantly increased glutathione peroxidase (GPx) activity and improved antioxidant capacity in cryopreserved ovarian transplants, thereby promoting tissue preservation and viability. Likewise, Damous et al.^24^ reported that scaffold-based melatonin delivery improved the structural integrity and survival of frozen–thawed ovarian autografts by reducing oxidative damage and supporting tissue regeneration. Furthermore, a recent systematic review by Monteiro et al.^25^ concluded that melatonin consistently exerts antioxidant effects in experimental animal models by scavenging ROS, suppressing lipid peroxidation, enhancing the activities of antioxidant enzymes including GPx, superoxide dismutase (SOD), and catalase (CAT), and modulating inflammatory signaling pathways. Although oxidative stress biomarkers were not evaluated in the present study, the histopathological improvements observed following green tea extract administration suggest that catechin polyphenols may exert protective effects through mechanisms similar to those described for melatonin. However, because biochemical markers of oxidative stress were not measured, any mechanistic similarity between green tea extract and melatonin remains speculative and should be interpreted with caution. Future experimental studies directly comparing these antioxidant agents while incorporating biochemical and molecular analyses are warranted to determine whether they share common cytoprotective pathways or exert distinct mechanisms of action during ovarian I/R injury.

The present study has several limitations. First, only a single dose and administration protocol of green tea extract were investigated. Dose-response studies are needed to determine the optimal therapeutic concentration and evaluate potential prooxidant effects associated with higher catechin doses. In addition, long-term ovarian reserve, hormonal profiles, and fertility outcomes were not assessed.

Second, although green tea extract significantly reduced histopathological ovarian injury, the concomitant increase in Bax and caspase immunoreactivity complicates the interpretation of the underlying cellular mechanisms. While this finding may reflect regulated apoptotic clearance of damaged cells during tissue repair, the present study was not designed to distinguish protective apoptosis from apoptosis associated with ongoing tissue injury. Additional mechanistic analyses, including terminal deoxynucleotidyl transferase dUTP nick-end labeling (TUNEL) assay, assessment of anti-apoptotic markers such as B-cell lymphoma-2 (Bcl-2), and evaluation of the Bax/Bcl-2 ratio, would have provided a more comprehensive characterization of apoptosis and its role in ovarian I/R injury.

Finally, a major limitation of the present study is the absence of biochemical assessments of oxidative stress and antioxidant defense. Although green tea extract significantly attenuated histopathological ovarian injury, key oxidative stress biomarkers, including malondialdehyde (MDA), GPx, CAT, and SOD, were not evaluated. Consequently, the proposed antioxidant mechanism underlying the observed protective effects cannot be directly confirmed and remains speculative.

The lack of these biochemical data limits the mechanistic interpretation of the findings and precludes direct correlation between histopathological improvement and changes in oxidative stress status. Future studies incorporating molecular, biochemical, and functional analyses are needed to clarify the mechanisms of action of green tea extract and to determine its therapeutic potential in ovarian I/R injury.

## Conclusion

The present experimental study demonstrated that green tea extract attenuated several histopathological features of ovarian I/R injury, including vascular congestion, edema, and follicular degeneration, suggesting a partial protective effect on ovarian tissue. Although the green tea-treated group showed lower overall injury scores than the saline-treated group, this difference did not reach statistical significance. Furthermore, the increased Bax and caspase expression observed following green tea extract administration indicates that green tea polyphenols may modulate apoptotic signaling pathways during reperfusion, although the underlying mechanisms remain unclear.

Because the underlying mechanisms were not directly investigated, these findings should be interpreted cautiously. Future studies incorporating oxidative stress biomarkers, comprehensive apoptotic pathway analyses, dose–response evaluations, and long-term reproductive outcomes are warranted to clarify the mechanisms and therapeutic potential of green tea-derived antioxidants in ovarian I/R injury.

## Data Availability

The datasets used or analyzed during the current study are available from the corresponding author on reasonable request.
